# Optimizing the Multimerization Properties of Quinoline-Based Allosteric HIV-1 Integrase Inhibitors

**DOI:** 10.3390/v16020200

**Published:** 2024-01-28

**Authors:** Jian Sun, Jacques J. Kessl

**Affiliations:** Department of Chemistry and Biochemistry, University of Southern Mississippi, Hattiesburg, MS 39406, USA

**Keywords:** HIV, integrase, virus maturation, allosteric integrase inhibitor, ALLINI, quinoline, aberrant integrase multimerization

## Abstract

Allosteric HIV-1 Integrase (IN) Inhibitors or ALLINIs bind at the dimer interface of the IN, away from the enzymatic catalytic site, and disable viral replication by inducing over-multimerization of IN. Interestingly, these inhibitors are capable of impacting both the early and late stages of viral replication. To better understand the important binding features of multi-substituted quinoline-based ALLINIs, we have surveyed published studies on IN multimerization and antiviral properties of various substituted quinolines at the 4, 6, 7, and 8 positions. Here we show how the efficacy of these inhibitors can be modulated by the nature of the substitutions at those positions. These features not only improve the overall antiviral potencies of these compounds but also significantly shift the selectivity toward the viral maturation stage. Thus, to fully maximize the potency of ALLINIs, the interactions between the inhibitor and multiple IN subunits need to be simultaneously optimized.

## 1. Introduction

The catalytic activity of the Integrase (IN) enzyme of the Human Immunodeficiency Virus type 1 (HIV-1) plays a major role during the early stage of the virus life cycle as it is responsible for the integration of the viral DNA (vDNA) into the host chromatin. This integration is composed of two successive but distinct events. In the first, IN removes a GT dinucleotide from both 3′ ends of the vDNA (termed 3′-processing or 3P). After the capture of a target DNA (tDNA), the strand transfer takes place where the vDNA recessed ends are inserted into the tDNA in a transesterification reaction. This second event has been successfully targeted by several FDA-approved inhibitors (Raltegravir, Elvitegravir, Dolutegravir, Bictegravir, and Cabotegravir) [1] that are currently used clinically to treat HIV-1 infected patients. Although these treatments are extremely effective, resistant strains have emerged against several of these drugs due to the high viral mutation rates [2,3]. Thus, these viral escape mutations within the active site of IN underline the importance of pursuing alternative mechanisms of inhibition with new binding sites on the enzyme.

The HIV-1 IN is structured into three distinct domains: N-terminal domain (NTD), catalytic core domain (CCD), and C-terminal domain (CTD) [4,5]. During integration, the three domains interact to form an ordered multimeric structure with the vDNA, the intasome [6,7,8,9,10]. Integration of the HIV-1 genome also involves the interaction between IN and the host chromatin-associated co-factor LEDGF/p75 (Lens Epithelium-Derived Growth Factor), which bridges the intasome to active genes [11,12,13,14,15,16]. LEDGF/p75 interacts with the intasome through its Integrase Binding Domain (IBD) by inserting a small loop into a well-defined pocket located at the IN CCD dimer interface [14,17].

Allosteric HIV-1 Integrase Inhibitors (ALLINIs) [18,19,20,21,22], which are also known as LEDGINs (LEDGF/p75 Inhibitors) [23], NCINIs (Noncatalytic Site Integrase Inhibitors) [24], or INLAIs (IN-LEDGF Allosteric Inhibitors) [25], selectively bind at this LEDGF/p75 IBD binding pocket, away from the IN catalytic site and potently inhibit HIV-1 replication. Importantly, these compounds retain full potency against clinical strains resistant to the FDA-approved IN catalytic inhibitors [23].

## 2. Discovery and Initial Optimization of the Quinoline-Based ALLINIs

**Figure 1:**
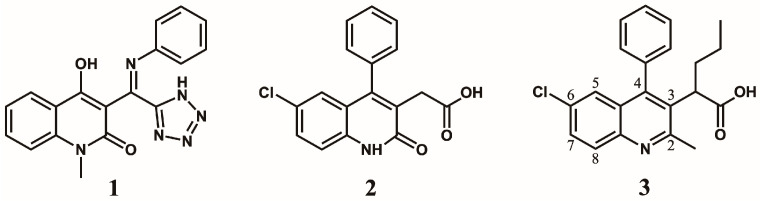
Quilonine-based inhibitors of IN-LEDGF/p75 interaction.

**Figure 2:**
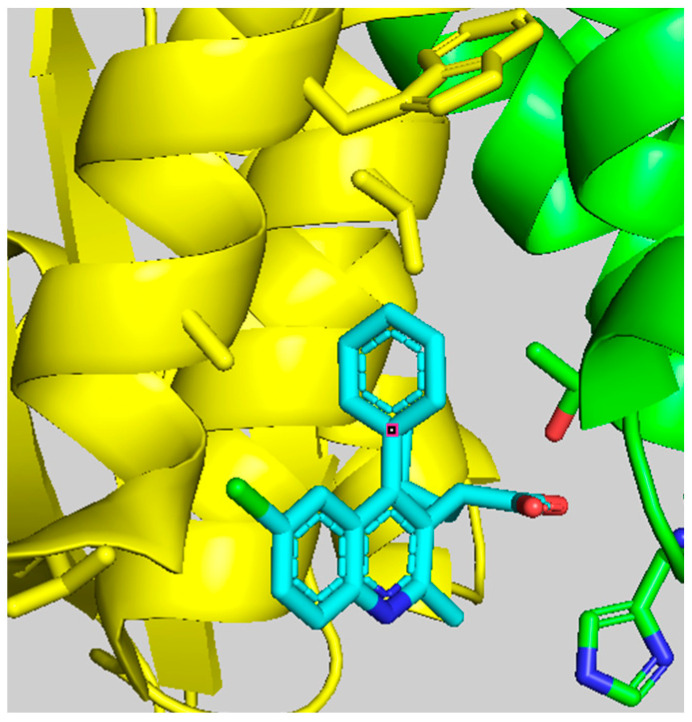
Crystal structure of quinoline **3** bound to IN CCD (pdb 3LPU).

The first quinoline-based ALLINIs were independently discovered by two distinct research groups at the University of Leuven (Belgium) [23] and at Boehringer Ingelheim (Canada) [26]. At Leuven, the initial hit compound **1** (Figure 1) was generated from a series of virtual screening calculations targeting the IBD binding pocket at the IN CCD dimer interface [23]. Testing of **1** confirmed an in vitro inhibition of the IN-LEDGF/p75 interactions by 36% at 100 μM using an AlphaScreen-based protein–protein binding assay [27]. Through several iterations, this hit was further refined into quinoline **2** (Figure 1) by replacing the tetrazole with carboxylic acid, removal of the unstable secondary ketimine at position 3, addition of a benzene group at position 4, and addition of a chlorine at position 6 (scaffold numbering shown in Figure 1). This improved quinoline was now able to inhibit the IN-LEDGF/p75 interactions in vitro with an IC_50_ of 12.2 ± 3.4 μM and showed weak antiviral activity (41.9 ± 1.1 μM). Further medicinal chemistry effort yielded quinoline **3** (also named LEDGIN-6) with an IN-LEDGF/p75 interaction IC_50_ of 1.37 ± 0.36 μM and an antiviral activity at 2.35 ± 0.28 μM. As the compounds of this series were optimized to prevent the binding of LEDGF/p75 to IN, they were termed LEDGINs. The binding of LEDGINs at the IBD binding pocket on the IN CCD dimer interface was confirmed via X-ray structure determination of IN CCD crystals soaked with quinoline **3** (Figure 2) [23].

**Figure 3:**
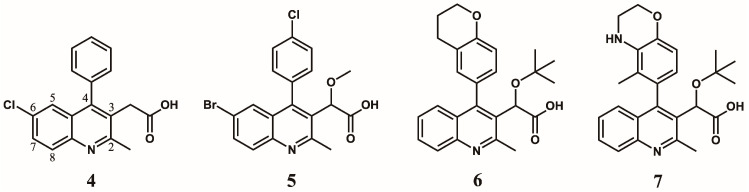
Quinoline-based inhibitors of 3P activity.

At Boehringer Ingelheim, a similar series of quinolines (Figure 3) with antiviral activities were identified through a high throughput screening (HTS) campaign targeting the 3P enzymatic activity of IN [26]. Testing of the initial hit compound **4** (Figure 3) showed an in vitro inhibition of the 3P catalytic function of IN at 9.0 μM using a FRET-based assay [28]. Medicinal chemistry efforts improved the series 300-fold to compound **5** (Figure 3, also named BI-B or BI-1001) with an IC_50_ in the same 3P assay at 28 nM. This improved quinoline was also able to inhibit viral replication with an EC_50_ at 450 nM. As for the LEDGINs, crystallographic studies confirmed the binding of the compound at the IBD binding pocket on the IN CCD dimer interface [26]. This crystal structure was used to optimize the series into **6** and **7** (Figure 3, also named BI-D and BI-C) [29] by adding a tert-butyl group at the alpha position of the carboxylic function, further filling up the binding pocket. Additional hydrophobic bulk was also added in position 2 of the quinoline scaffold, improving the 3P IC_50_ into the single-digit nanomolar range at 6 nM and 3 nM, respectively, and bringing the antiviral activity EC_50_ at 10.0 nM [29] and 4.2 nM [26], respectively.

## 3. Integrase Multimerization by ALLINIs

**Figure 4:**
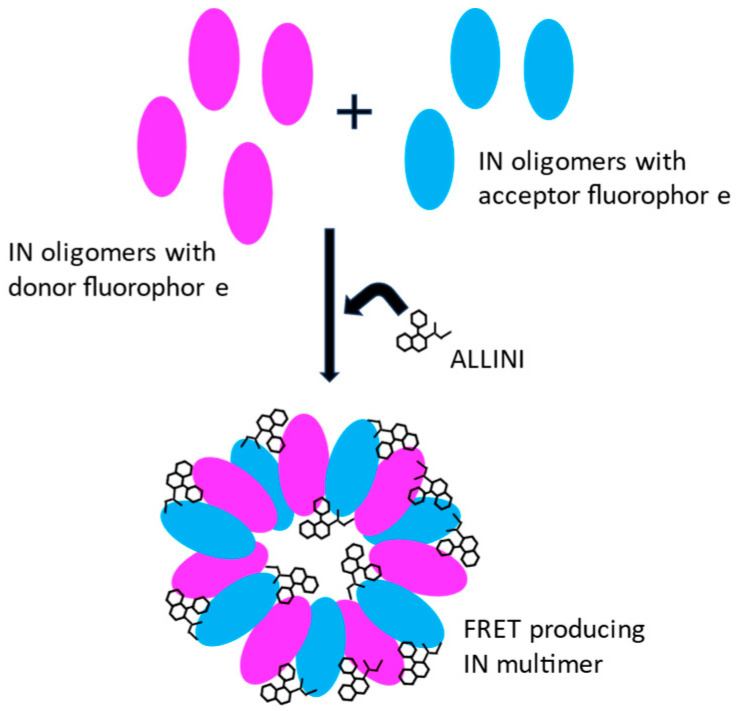
FRET-based IN multimerization in vitro assay.

In order to reconcile the different types of activities observed with the two quinoline series described above, comparative studies with representative compounds **3** (LEDGIN-6) and **5** (BI-1001) were initiated by the Kvaratskhelia group (The Ohio State University, USA) [18]. This work was the first to propose that the inhibition of both IN-LEDGF/p75 interaction and IN catalytic functions were likely a secondary outcome of the mode of action of those compounds toward the IN protein: a strong and rapid induction of IN hyper-multimerization. This effect was initially observed in vitro using a FRET-based assay and recombinant IN [18,30] (Figure 4). This ALLINI-induced hyper-multimerization was shown to render IN incapable of binding to LEDGF/p75 or performing its normal catalytic functions. In addition, the in vitro compounds inhibitory concentrations were similarly effective in blocking overall antiviral replication in cell culture [18]. These observations were subsequently confirmed using similar assays and compounds [24,31].

While the initial studies expected that the potency of these compounds was during the integration step, subsequent observations clarified this as a secondary consequence of the IN hyper-multimerization. The Engelman group (Harvard Medical School, USA) was the first to measure ALLINI potencies during HIV-1 egress (where the infectivity of viral particles produced from ALLINI-treated cells was assessed in untreated target cells) versus HIV-1 ingress (where target cells were treated with ALLINIs during infection). This approach revealed that ALLINIs inhibited the late replication stage more potently (10 to 100-fold or more, depending on the compound) than the early stage [32]. These observations were independently confirmed and published during the same year using similar assays and compounds [31,33,34]. Observations of the morphology of viral particles produced from ALLINI-treated cells have shown morphological defects where the viral ribonucleoprotein complex (RNP) is mis-localized to an eccentric position between the empty capsid core and the virion’s matrix layer instead of being properly encapsulated into the core [32]. Thus, these observations have led to the hypothesis that IN plays a critical non-catalytic role during viral maturation that can be uniquely targeted using ALLINIs. Subsequently, the Kvaratskhelia group (Ohio State University, USA) demonstrated that ALLINI-induced IN-hyper-multimerization inhibited viral RNA binding, explaining the observed effects of these compounds on virion morphology [35].

## 4. Binding Features of Quinoline-Based ALLINIs

**Figure 5:**
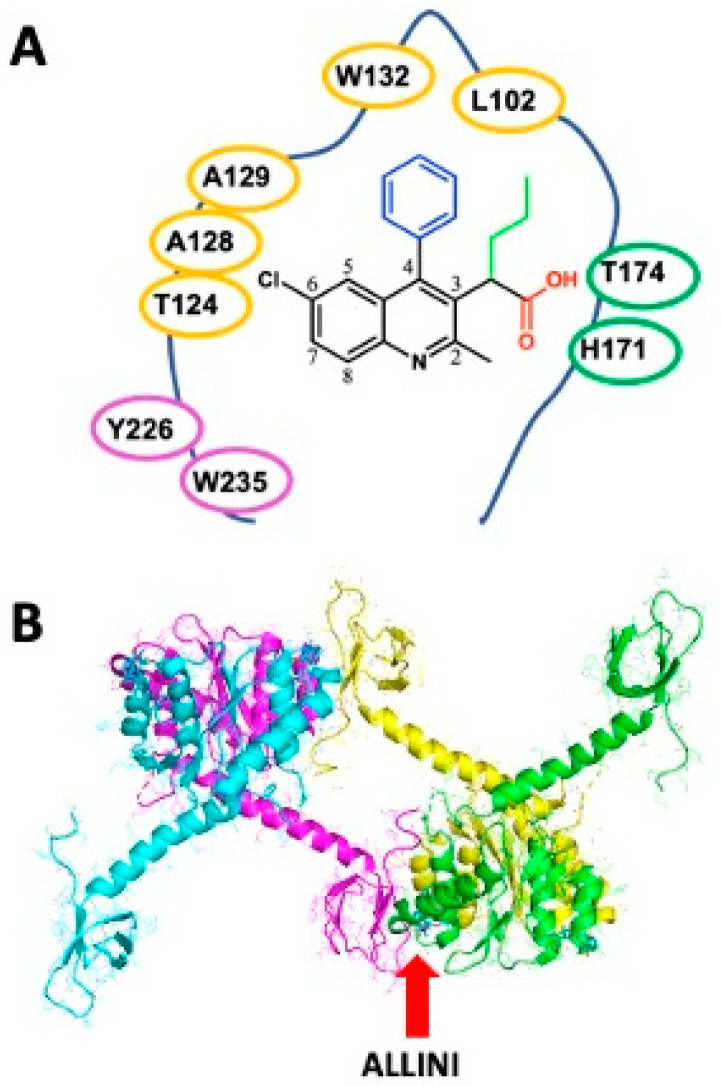
ALLINI binding features. ALLINI binding pocket (**A**) and IN subunit crystallographic arrangement (**B**). The IN subunits 1, 2, and 3 are color-coded green, yellow, and magenta, respectively (pdb 5HOT).

As described above, ALLINIs bind at the LEDGF/p75 binding pocket and engage the IN CCD dimer interface at a position distal from the enzyme active site. Early X-ray structures of IN CCD crystals soaked with LEDGINs [23] (Figure 2) were able to provide valuable information on the binding topology. Quinoline-based ALLINIs harbor several essential features, including a branched aliphatic group ending with a carboxylic acid function in position 3 (green and red groups on the quinoline in Figure 5A) and a large aromatic substitution in position 4 (blue substitution on the quinoline in Figure 5A). This large aromatic group can be optimized to interact with W132 and L102 of subunit 2 (yellow residues in Figure 5A). The aliphatic side chain (green chain on the quinoline in Figure 5A) buries into the CCD-CCD interface, where it contacts hydrophobic amino acids from both subunits 1 and 2 of the IN dimer. The carboxylic acid group (red) interacts with H171 and T174 of the IN subunit 1. This interaction recapitulates the binding mode of LEDGF/p75, explaining why many ALLINIs (or LEDGINs) are effective inhibitors of the IN-LEDGF/p75 interaction.

Through computer simulations [36,37,38], crystallography [39,40,41,42], and biochemical experiments [37,38,43,44], it has been shown that in addition to binding at the IN CCD dimer interface of subunits 1 and 2, ALLINIs also bridge with the CTD of a third IN subunit (subunit 3, color-coded magenta in Figure 5A,B) to form a polymer-like repetitive pattern alternating ALLINIs and IN monomers. Thus, quinoline-based ALLINIs binding to the IN oligomer and antiviral properties have both been improved by extending the reach of the compound toward Y226 and W235 of the third IN subunit (Figure 5A) [37,38].

## 5. Optimization of the Quinoline-Based ALLINIs

Multiple studies using quinoline-based ALLINIs have shown that the antiviral potencies of these compounds, especially at the late phase, are tightly correlated with their in vitro multimerization properties [18,24,30,32,37,38]. Thus, this review will use the published IN multimerization EC_50_ of compounds to compare and rank the effects of various substitutions attached at different positions on the quinoline scaffold. When available, the measured antiviral activities will also be discussed.

### 5.1. Optimization of Position 4

Substitutions added to position 4 of the quinoline scaffold aim to maximize interaction between the compound and the hydrophobic pocket defined by the residue pair W132/L102 of subunit 2 (yellow residues in Figure 5A). Thus, compounds with aromatic groups on position 4 are highly favored, with a preference for substituted phenyl groups [22]. Extensive exploration of the chemical space demonstrates that several single para substitutions of the phenyl group (Table 1) improve the compound’s multimerization EC_50_s compared with the unsubstituted derivative **8**. Substitutions with fluorine (compound **10**) or fluorine-containing groups such as trifluoromethyl and trifluoromethoxy (compounds **12** and **14**) slightly decrease EC_50_s by 2–4 folds while substitution for either a methyl or a methoxy group (compounds **11** and **13**) resulted in a six-fold improvement in the EC_50_ values. Interestingly, adding a chlorine (compound **9**) produced the most effective inhibition among this series with an EC_50_ of 100 nM [22], which resulted in a 13-fold improvement compared with the unsubstituted phenyl group. This strong effect was attributed to the stabilizing effect of the chlorine−π interaction provided by the side chain of W132 [45]. Larger substitutions such as cyano (**16**), acetyl (**17**), acetamido (**18**), or phenyl (**19**), were found to have a negative contribution to the EC_50_.

Meta- or ortho-substituted phenyl additions were shown to be less effective than para counterparts (Table 2) in improving the compounds [22]. The meta and ortho positions place the substituted group away from the W132/L102 pair (Figure 5A), which attenuates the hydrophobic interactions. For most cases with the same R group, the meta position resulted in the worse potency producing the trend: para > ortho > meta [22]. Attempts to combine para and meta (same R) into disubstituted phenyl groups did not result in significant EC_50_ improvement. The measurements were either similar to the para alone (compounds **11** vs. **32**) or worse (compounds **13** and **23** vs. **33**) [22,24].

**Figure 6:**
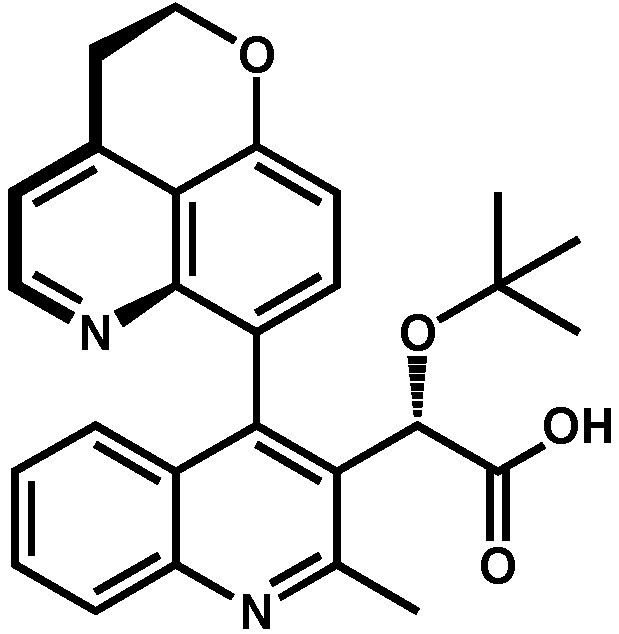
BI 224436.

Because the phenyl group (compound **8**) is able to fill the binding pocket more efficiently than smaller pentadiene-like groups such as furane or thiophene (compound **8** vs. **34** and **35**, Table 3), larger ring systems are more likely to achieve a greater degree of multimerization. It was found very early in the development of these quinolines by Boehringer Ingelheim that adding a chromane group in position 4 (compound **6** in Table 3, also named BI-D) was effective in bringing both in vitro IN multimerization EC_50_ and antiviral potency in the tenth of nanomolar range. Additional variations, such as benzodioxane (compound **36**) or substituted benzoxane (compound **37**), confirmed the effect. Interestingly, it was observed that the addition of substitutions on the benzoxane ring system (such as on compounds **7** and **37**) restricted the free rotation of the group and improved the antiviral potencies even further [46]. This led to the development of the tricyclic BI 224436, which completely blocked the rotation and allowed the synthesis of stable atropoisomers [47]. Further testing showed that the stereoisomer displayed in Figure 6 had antiviral activities in the single-digit nanomolar range [46]. Additionally, this compound was found to display appropriate metabolic stability in liver microsomal oxidation assay and was further evaluated in several animal models (mouse, rat, dog, and monkey) for preclinical profiling [48]. Thus, BI 224436, which exhibited excellent pharmacokinetic and toxicologic properties in animals, was successfully advanced into clinical development [48].

### 5.2. Optimization of Position 6

Because of its presence on position 6 of the initial HTS hit compound **4**, the chlorine was thought to be important in anchoring the scaffold in the IBD pocket as it interacts with the triad A129/A128/T124 of IN subunit 2 (Figure 5A). Early optimization efforts replaced the chlorine with a bromine and improved this interaction slightly [46]. The effect of adding this bromine on position 4 can be observed by comparing the EC_50_s of **9** and **39** (Table 4).

Nevertheless, substitution with iodine (**40**) or amino (**41**) groups was tested and resulted in a two-fold decrease in the EC_50_ values (Table 4) [38]. To probe for additional interactions with A128 and A129, a library of quinolines with position 6-substituted phenyl groups (Table 4) was generated. Testing of several factors including aromatic interactions that may contribute to the binding showed that the addition of aryl groups on position 6 negatively impacted the multimerization properties of the scaffold. Further increases in the measured EC_50_ were observed with substituted phenyl groups (compare compound **42** with compounds **43** to **50** in Table 4). Additional groups, such as pyridinyl (**51**), non-aromatic six-membered heterocycles (**52** and **53**), and five-membered aromatic heterocycles (**54–57**) were also tested. It was found that a significantly lower potency, or the complete loss of activity, was caused by the added bulk [38].

Experiments conducted at Gilead Sciences, Inc. looking for the selection of drug-resistance against ALLINIs such as **32** and **37** revealed the IN mutation A128T as one of the most frequent occurrences [24]. Using compound **39,** which has bromine in position 6, the structural and mechanistic basis for this effect was subsequently elucidated by showing that the A128T substitution significantly shifted the positioning of the inhibitor in the binding pocket [19]. As marginal improvements in EC_50_s can be obtained via substitution at position 4, those gains must be balanced with the possibility that they may magnify the resistance amplitude when the IN mutation A128T emerges.

### 5.3. Optimization of Position 7

In order to leverage recent discoveries showing that ALLINIs also bridge with the CTD of a third IN subunit (subunit 3, color-coded magenta in Figure 5A,B) and form a polymer-like repetitive pattern, modifications at position 7 were explored [37]. These substitutions aim to improve the multimerization properties of ALLINIs by extending their reach toward Y226 and W235 of the third IN subunit (Figure 5A).

**Figure 7:**
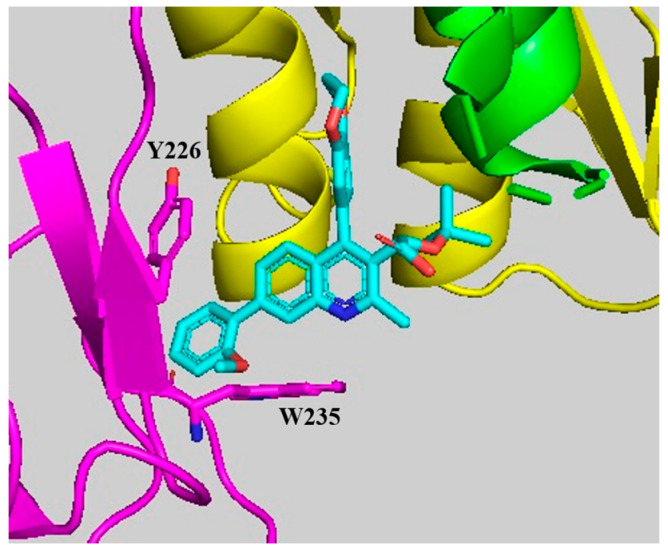
Binding model of compound **66**.

The compound **60,** which added a simple phenyl group at position 7, showed a slight improvement in the multimerization EC_50_ value compared with the unsubstituted compound **36** [37]. Seeking additional hydrophobic interactions with W235, a variety of ortho-substitutions on the phenyl ring were screened (compounds **61–66**). Among those, three compounds displayed better EC_50_ values than **60**, with **66** having the lowest multimerization EC_50_ (Table 5). To further explore the properties of this series, both the meta (**67**) and the para (**68**) methoxy variants were tested. While the para substitution (**68**) displayed a multimerization EC_50_ of 0.04 μM like the ortho (**66**), the meta variant (**67**) was notably less potent (Table 5) [37]. To further confirm the significant contribution of manipulating position 7, the IC_50_ late-stage antiviral values of **36**, **60,** and **66** were measured in parallel as 0.66 μM, 0.05 μM, and 0.01 μM, respectively [37]. Analytical sucrose density gradient experiments revealed that treatment of producing cells with **66** resulted in a significant shift of the viral capsid core density toward lower values. These results indicate that the density of the viral cores decreased upon ALLINI treatment and are consistent with the formation of an empty core caused by the mislocalization of the viral ribonucleoprotein [20,35,37]. Using previously obtained crystal structures [39,40], a computer-based binding model of compound **66** was generated and showed that the ortho-substituted benzyl group on position 7 maximized pi–pi interactions with the aromatic residues Y226 and W235 of the third subunit (Pink subunit, Figure 7) [37]. This additional interaction between the IN CCD and CTD bridged by the ALLINIs was measured using a FRET-based assay that combined full-length recombinant IN and CTD fragments [37].

### 5.4. Optimization of Position 8

Additional attempts to bridge toward the CTD of the third IN subunit were made by substituting position 8 of the quinoline scaffold [38] ever with the chlorophenyl or the benzodioxane in position 4. It was found that the addition of bromine (compounds **69** and **75**) slightly improved the observed in vitro EC_50_ for multimerization (Table 6). With the objective of promoting further hydrophobic interactions between the compound and the Y226-W235 residues of the CTD, a phenyl group (**70**) and ortho, meta, para 8-methoxyphenyl-substituted analogs (**71**–**73**) were tested. It was measured that contrary to the improvement observed with position 7, the addition of these aryl groups on position 8 negatively impacted the multimerization properties of the compounds [38]. The late-stage antiviral activities of **36** and **75** were measured in parallel in both WT and A128T constructs. Interestingly, it was found that while **36** had similar IC_50_s for both constructs, compound **75** was two-fold more potent in the A128T (0.06 μM for WT vs. 0.03 μM for A128T). Molecular modeling of the binding of these two compounds in both WT and A128T structures revealed that the T128 mutation shifts the inhibitor slightly out of the binding pocket, even without any substitution in position 6 (see above). This push is calculated to slightly increase the weak pi–Br interaction between the aromatic side-chain of W235 and compound **75** [38].

## 6. Discussion

Current CDC guidelines (www.cdc.org, accessed on 1 January 2024) for first-line antiretroviral therapy for people infected with HIV-1 must include one of the FDA-approved IN inhibitors (Raltegravir, Elvitegravir, Dolutegravir, Bictegravir, and Cabotegravir). As viral escape mutations have emerged at the active site of IN where these drugs bind, new potent IN inhibitors with alternative inhibition mechanisms are needed. The ALLINIs (or Allosteric HIV-1 Integrase Inhibitors) bind at the LEDGF/p75 IBD binding pocket, away from the IN catalytic site and potently inhibit HIV-1 replication [23]. Upon binding to their target, they induce a strong and rapid IN hyper-multimerization that disables its functions [18]. Quinoline-based ALLINIs have been shown to be more potent during the late replication stage as IN hyper-multimerization interferes with the viral RNA-IN binding step during HIV-1 maturation causing late-stage effect and particle morphological defects [32,35]. As these late-phase replication effects are tightly correlated with the IN multimerization properties of these compounds, FRET-based in vitro assay has been used by several groups to rank and optimize this class of inhibitors [18,24,31,34].

Early development of the scaffold by Boehringer Ingelheim has shown that the tert-butoxy acetic acid side chain in position 3 was optimal as it provides essential hydrogen bond interactions with the T174 and H171 residues, mimicking the LEDGF/p75 binding pattern [46]. Thus, this review has focused on the published optimization efforts on the quinoline scaffold by examining the effects of various substitutions attached at positions 4, 6, 7, and 8.

Substitutions added to position 4 maximize the interaction between the compound and the hydrophobic pocket defined by the residue pair W132/L102 [22,46]. After position 3, substitutions at this location have the biggest impact on the compound properties. Substituted aromatic groups such as chlorobenzyl, mono, or dioxane were very effective in improving both IN multimerization EC_50_s and antiviral IC_50_s [22,46]. Further modifications aiming to restrict the free rotation of the group at the C4 bond improved the antiviral potencies even further and led to the synthesis of BI 224436 [46]. This Boehringer Ingelheim compound has been shown to display excellent pharmacokinetic and toxicologic properties and has advanced into clinical trials [48].

Large substitutions at position 6 negatively impacted the multimerization properties of the compounds. As the left edge of the unsubstituted scaffold is already close enough to establish hydrophobic interaction with the triad A129/A128/T124, anything bigger than a bromine was measured to be too bulky. Additionally, drug resistance studies have revealed the IN mutation A128T, which is capable of using such substitution to leverage the ALLINI out of the pocket. Thus, the position should be left unsubstituted [38].

Substitutions at positions 7 and 8 both aim to improve the multimerization properties of ALLINIs by extending their reach toward Y226 and W235 of the third IN subunit. Thus, adding an ortho-substituted phenyl group at position 7 or bromine at position 8 was effective in improving both IN multimerization EC_50_s and antiviral IC_50_s, hinting that additional contact points with IN could be achieved with these additions [37,38]. These measurements suggest it could be interesting to test the substitution of these two positions in tandem.

## 7. Conclusions

The discovery and development of quinoline-based ALLINIs is a perfect example of complementarity and synergy between research in academia and the pharmaceutical industry. As both Boehringer Ingelheim and Gilead Sciences, Inc. were very effective in developing the initial HTS hits into potent compounds with excellent pharmacological properties, academic research groups, intrigued by the unexpected properties of these compounds, became key players in unmasking their true mode of actions. Detection of the IN multimerization properties of those compounds was followed by the discovery of their potency at a late stage, which eventually led to the observation of their role in inhibiting the vRNA-IN interactions. As the latter of those effects seems to be an indirect consequence of the drug-induced aberrant multimerization of IN, those breakthroughs may open a new frontier in HIV-1 drug discovery: the search for a novel class of inhibitors capable of directly blocking the IN-vRNA interaction.

## Figures and Tables

**Table 1:** Optimization of position 4.

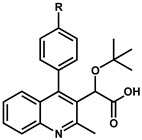					
Compound	R	EC_50_ (µM) ^a,b^	Compound	R	EC_50_ (µM) ^a,b^
**8**	H	1.32 ± 0.53	**14**	OCF_3_	0.35 ± 0.09
**9**	Cl	0.10 ± 0.02	**15**	SCH_3_	0.49 ± 0.01
**10**	F	0.49 ± 0.04	**16**	CN	2.97 ± 0.81
**11**	CH_3_	0.24 ± 0.11	**17**	COCH_3_	1.39 ± 0.48
**12**	CF_3_	0.72 ± 0.06	**18**	NHCOCH_3_	8.41 ± 0.73
**13**	OCH_3_	0.23 ± 0.04	**19**	Phenyl	no activity

^a^ Multimerization assay; ^b^ data from [22].

**Table 2:** Optimization of position 4.

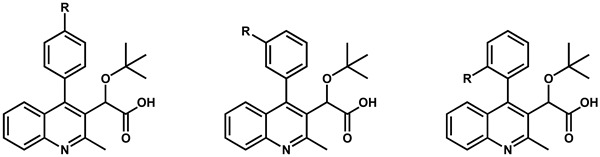						
R-para		R-meta			R-ortho	
R group	Compound (R-para)	EC_50_ (µM) ^a,b^	Compound (R-meta)	EC_50_ (µM) ^a,b^	Compound (R-ortho)	EC_50_ (µM) ^a,b^
Cl	**9**	0.10 ± 0.02	**20**	3.79 ± 0.09	**26**	0.26 ± 0.04
F	**10**	0.49 ± 0.04	**21**	2.11 ± 0.59	**27**	0.58 ± 0.07
CH_3_	**11**	0.24 ± 0.11	**22**	0.95 ± 0.29	**28**	0.51 ± 0.05
OCH_3_	**13**	0.23 ± 0.04	**23**	1.38 ± 0.37	**29**	8.39 ± 0.92
SCH_3_	**15**	0.49 ± 0.01	**24**	1.00 ± 0.14	**30**	4.51 ± 0.71
CN	**16**	2.97 ± 0.81	**25**	no activity	**31**	11.14 ± 0.85

^a^ Multimerization assay; ^b^ data from [22].

**Table 3:** Optimization of position 4.

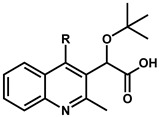					
Compound	R	EC_50_ (µM) ^a,b^	Compound	R	EC_50_ (µM) ^a,b^
**32**	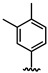	0.25 ^c^	**6**	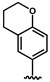	0.03 ± 0.01 ^d^
**33**	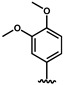	3.70 ± 0.90	**36**	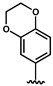	0.08 ± 0.01
**34**	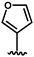	2.21 ± 0.56	**37**	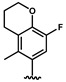	0.02 ^c^
**35**	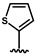	no activity	**38**	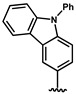	no activity

^a^ Multimerization assay; ^b^ data from [22] unless indicated; ^c^ data from [24], no SD available; ^d^ data from [32].

**Table 4:** Optimization of position 6.

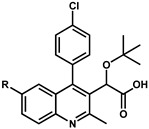								
Compound	R	EC_50_ (µM) ^a,b^	Compound	R	EC_50_ (µM) ^a,b^	Compound	R	EC_50_ (µM) ^a,b^
**39**	Br	0.09 ± 0.01 0.07 ± 0.01 ^c^	**46**	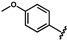	1.28 ± 0.01	**53**	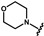	no activity
**40**	I	0.20 ± 0.06	**47**	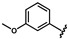	1.30 ± 0.37	**54**	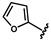	1.08 ± 0.17
**41**	NH_2_	0.19 ± 0.05	**48**	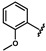	1.20 ± 0.14	**55**	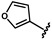	no activity
**42**	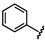	1.17 ± 0.14	**49**	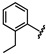	no activity	**56**	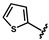	1.53 ± 0.13
**43**	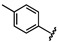	1.59 ± 0.04	**50**	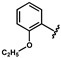	no activity	**57**	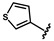	1.53 ± 0.33
**44**	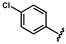	1.93 ± 0.01	**51**	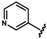	0.57 ± 0.29	**58**	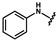	no activity
**45**	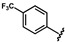	1.29 ± 0.01	**52**	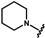	0.97 ± 0.40	**59**	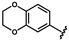	no activity

^a^ Multimerization assay; ^b^ data from [38]; ^c^ data from [19].

**Table 5:** Optimization of position 7.

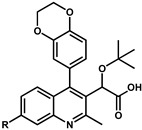					
Compound	R	EC_50_ (µM) ^a,b^	Compound	R	EC_50_ (µM) ^a,b^
**36**	H	0.08 ± 0.01	**64**	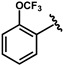	0.12 ± 0.01
**60**	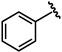	0.07 ± 0.01	**65**	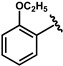	0.06 ± 0.01
**61**	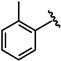	0.06 ± 0.01	**66**	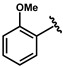	0.04 ± 0.01
**62**	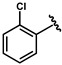	0.11 ± 0.01	**67**	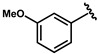	0.52 ± 0.01
**63**	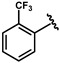	0.14 ± 0.01	**68**	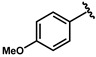	0.04 ± 0.01

^a^ Multimerization assay; ^b^ data from [37].

**Table 6:** Optimization of position 8.

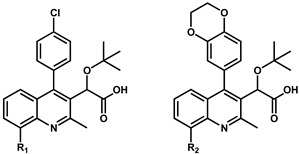								
Compound	R_1_	EC_50_ (µM) ^a,b^	Compound	R_1_	EC_50_ (µM) ^a,b^	Compound	R_2_	EC_50_ (µM) ^a,b^
**9**	H	0.10 ± 0.02	**71**	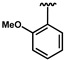	0.26 ± 0.04	**36**	H	0.08 ± 0.01
**69**	Br	0.09 ± 0.01	**72**	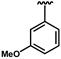	0.24 ± 0.01	**74**	Cl	0.08 ± 0.01
**70**	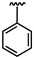	0.28 ± 0.03	**73**	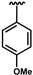	0.35 ± 0.08	**75**	Br	0.05 ± 0.01

^a^ Multimerization assay; ^b^ data from [38].
